# Pathogenic Role of IL-17 and Therapeutic Targeting of IL-17F in Psoriatic Arthritis and Spondyloarthropathies

**DOI:** 10.3390/ijms241210305

**Published:** 2023-06-18

**Authors:** Guillermo Sánchez-Rodríguez, Lluís Puig

**Affiliations:** Department of Dermatology, Hospital de la Santa Creu i Sant Pau, Universitat Autònoma de Barcelona, Carrer de Sant Quintí, 89, 08041 Barcelona, Spain; gsanchezr@santpau.cat

**Keywords:** psoriatic arthritis, axial spondyloarthritis, interleukin 17F, psoriasis, bimekizumab, sonelokimab

## Abstract

The interleukin 17 (IL-17) family, a subset of cytokines consisting of IL-17A-F, plays crucial roles in host defence against microbial organisms and the development of inflammatory diseases, including psoriasis (PsO), axial spondyloarthritis (axSpA), and psoriatic arthritis (PsA). IL-17A is the signature cytokine produced by T helper 17 (Th17) cells and is considered the most biologically active form. The pathogenetic involvement of IL-17A in these conditions has been confirmed, and its blockade with biological agents has provided a highly effective therapeutical approach. IL-17F is also overexpressed in the skin and synovial tissues of patients with these diseases, and recent studies suggest its involvement in promoting inflammation and tissue damage in axSpA and PsA. The simultaneous targeting of IL-17A and IL-17F by dual inhibitors and bispecific antibodies may improve the management of Pso, PsA, and axSpA, as demonstrated in the pivotal studies of dual specific antibodies such as bimekizumab. The present review focuses on the role of IL-17F and its therapeutic blockade in axSpA and PsA.

## 1. Introduction

**Figure 1:**
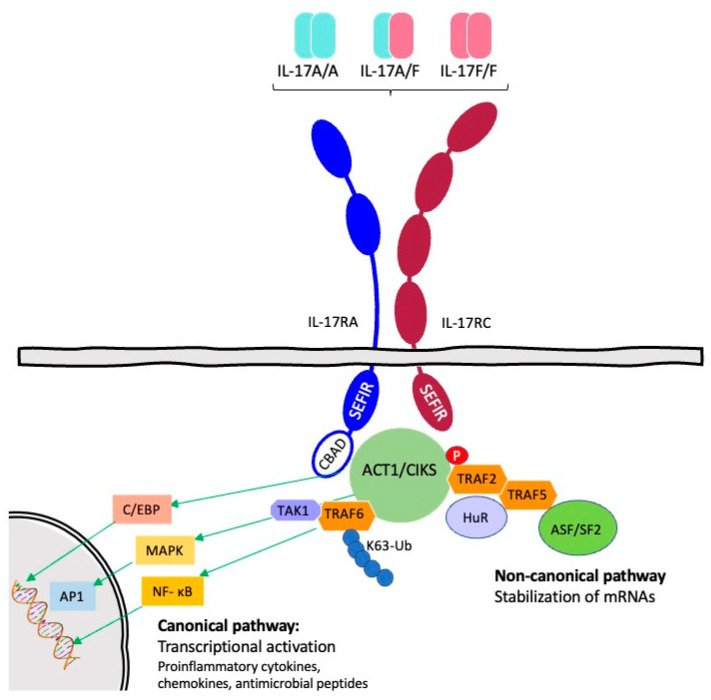
IL-17RA/RC signalling pathways (Figure adapted from Monin and Gaffen [3], and from Chung [4]). Homotypic interactions between the SEF/IL-17R (SEFIR) domains of the receptor and the adaptor Act1/CIKS are made possible by ligand (IL-17A/IL-17F/IL-17A/F) binding to the receptor complex. The nuclear factor kB (NF-kB) route, the CCAAT/enhancer-binding protein b (C/EBPb), and the mitogen-activated protein kinase (MAPK) pathways are all activated by the conventional IL-17 signalling pathway, which is started by Act1-induced K63 linked ubiquitination of TNF receptor associated factor 6 (TRAF6). Downstream target genes, such as those encoding proinflammatory cytokines, chemokines, and antimicrobial peptides, are then transcriptionally activated as a result. In contrast, noncanonical signalling relies on Act1 phosphorylation at amino acid 311, leading to recruitment of the RNA-binding protein HuR and formation of the TRAF2/TRAF5 complex, which sequesters the alternative splicing factor ASF/SF2 (mRNA-destabilizing) [3,4].

The interleukin 17 (IL-17) family consists of a subset of cytokines that participate in both acute and chronic inflammatory responses. Since the discovery of IL-17A in 1993, five other members of this family have been identified based on amino acid sequence homology and named sequentially (IL-17B to IL-17F). Human IL-17A and IL-17F map to 6p12.2 and are, with 55% amino acid sequence identity, the two most closely related members. All IL-17 family members exert their functions as disulphide-linked homodimers, except for IL-17A and IL-17F, which can also form heterodimers. IL-17A, IL-17F, and IL-17A/F signalling requires the formation of a heterotrimeric receptor complex comprising IL-17RA and IL-17RC. Both IL-17RA and IL-17RC are type I membrane receptors with an intracellular SEFIR signalling domain. Initial signalling events depend on homotypic interactions with the SEFIR motif of the adaptor protein Act1, leading to the TRAF6-dependent activation of the NF-κB and MAP kinase pathways. (Figure 1) [1,2,3,4].

Human IL-17RA shows different affinities for human IL-17A, IL-17F, and IL-17A/F. In comparison to IL-17A, IL-17F binds with at least 100-fold lower affinity to IL-17RA but with equally high affinity to IL-17RC; the IL-17A/F heterodimer shows intermediate binding affinity to IL-17RA, and the biological potencies of these three IL-17 cytokines in cell-based assays correlate with their binding affinities toward the IL-17RA receptor. IL-17A, the best known of these cytokines, has been linked to the pathogenesis of autoimmune and chronic inflammatory diseases, and its blockade with monoclonal antibodies has proved efficacious in psoriasis (PsO), psoriatic arthritis (PsA), and axial spondyloarthritis (axSpA).

Cells from both the innate and adaptive immune systems produce IL-17A and IL-17F, which stimulate target cells to synthesize and release inflammatory mediators such as tumour necrosis factor (TNF); IL-1; IL-6; granulocyte colony-stimulating factor (G-CSF); granulocyte-macrophage colony-stimulating factor (GM-CSF); antimicrobial peptides (AMPs); and chemokines such as CXCL1, CXCL5, CCL2, and CCL7 [1,2].

Th17 cells are the best-known producers of IL-17A and IL-17F, and IL-23 is particularly important in maintaining their differentiation state [5]. Nevertheless, recent studies show the existence of other cellular subtypes that can also produce IL-17A and IL-17F in an IL-23-independent manner—in addition to the IL-23 classical pathway [6,7,8,9,10,11].

The therapeutic targeting of individual cytokines of the so-called IL-23/IL-17 axis has proved efficacious in several immune-mediated inflammatory diseases (IMIDs), including PsO, PsA, axSpA, inflammatory bowel disease (IBD), and HS. Not all IMIDs respond equally to the blockade of IL-17 or IL-23; both approaches are similarly effective in PsO, whereas anti-IL-12/23 (ustekinumab) and anti-IL-23p19 monoclonal antibodies are inefficacious in axSpA, as opposed to IL-17A inhibitors, which are approved for this indication. Conversely, in IBD, both ustekinumab and anti-IL-23p19 antibodies are effective, whereas clinical trials of anti-IL17A agents have demonstrated a lack of efficacy and even worsening in some cases [1,2,3,4,12]. This has been attributed to the physiologic role of IL-17A in maintaining the integrity of the intestinal epithelium and protecting against some infections; on the other hand, the suppression of IL-17F, but not of IL-17A, provides protection against colitis by inducing Treg cells in a mouse model, suggesting that IL-17A and IL-17F may play distinct roles in the digestive tract [12].

Although several breakthrough discoveries on the importance of the IL-17 family have been reported, the complete identification of the role of IL-17F in health and disease balance remains elusive. The present review focuses on the role of IL-17F and its therapeutic blockade in PsA and axSpA.

## 2. Materials and Methods

The PubMed database (https://www.pubmed.gov, accessed on 1 April 2023) was used for this narrative review of the literature. The search string used was: “(interleukin 17F OR IL-17F) AND (psoriatic arthritis OR spondylitis OR spondyloarthritis)”, yielding 798 results. We analysed the abstract of each reference whose title suggested an association between IL-17F, PsO, PsA, and axSpA. The entire article was read only if the abstract indicated that the article potentially met our inclusion criteria: English language, research paper, human populations only, relevant to the outcome of interest, and/or full text available. Papers identified from the title, abstract, or full text as irrelevant to the topic were discarded.

## 3. IL-17 Family: Synthesis, Regulation, and Receptors

In the early 2000s, Th17 cells were discovered as a T helper (Th) subset distinct from Th1 and Th2 cells and characterized by the production of IL-17A. Five other members of the IL-17 family have been identified and given the designations IL-17B, C, D, E, and F. IL-17F is commonly generated by the same cell types as IL-17A and shares the highest degree of sequence homology (55%) within the family. The first member of the IL-17 family whose crystallographic structure was determined was IL-17F [3,13].

IL-17A is released as a disulphide-linked homodimeric glycoprotein with a dimer molecular weight of 30–35 kDa. IL-17B, C, and E all share a conserved C-terminal domain but have unique N-terminal portions [13]. The biological profiles of IL-17B, C, and E are very different from those of IL-17A. For example, IL-17B is expressed in the stomach, small intestine, and pancreas, whereas IL-17C is rarely expressed, and none of the three homologs are expressed by activated T cells [13].

Like IL-17A, IL-17F is released by activated T cells, stimulates the production of cytokines, and may play a significant role in the inflammation and skeletal damage associated with inflammatory arthritis. While the effects of IL-17F seem to be connected to those of IL-17A, its potency varies, which is consistent with variations in receptor-binding affinities [1,14].

**Figure 2:**
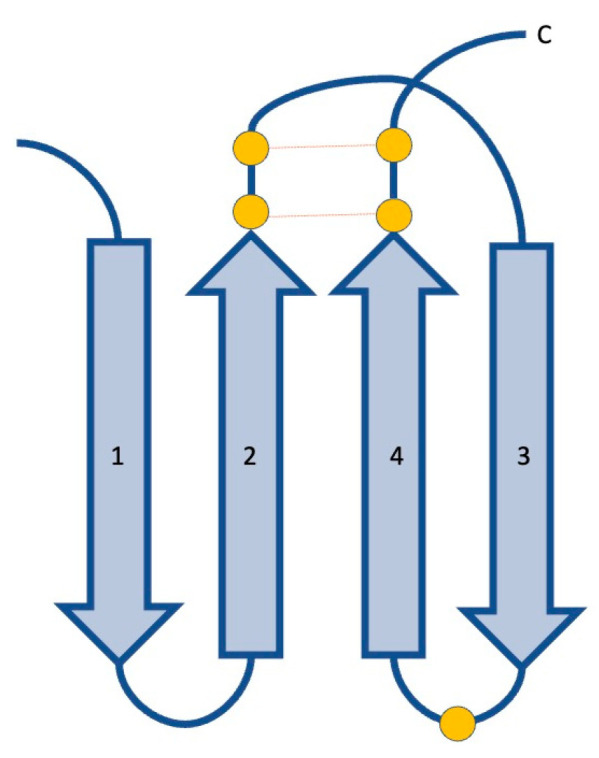
Cartoon representation of the structure of IL-17F. IL-17F is a structural homolog of proteins containing a cystine knot fold, characterized by two sets of paired β-strands (numbered 1–4) connected by disulphide linkages between strands 2 and 4, and a third disulphide bridge connecting strands 1 and 3 that is lacking in the IL-17F protomer *(Figure adapted from Hymowitz* [13]*). Cysteine residues are represented as orange circles, disulphide linkages as dashed lines of the same colour*.

The core of the IL-17F protomer is composed of two pairs of antiparallel β-strands: one pair includes strands 1 and 2, while the other includes strands 3 and 4. Strand 2 is interrupted by a short stretch of irregular β-structure. Two disulphide bridges connect strands 2 and 4. A third disulphide linkage connects the loop between strands 3 and 4 of one protomer to the N-terminal extension of the adjacent monomer forming extensive dimer contacts. This N-terminal extension (residues 1–48) also contains a β-strand and a small α-helix (Figure 2) [13].

All the family members except IL-17D, which binds to CD93, are functional as homodimers, but IL-17A and IL-17F also form a heterodimer. Except for the IL-17D receptor CD93, all IL-17 receptors form dimers after ligand binding. The first receptor identified for the IL-17A homodimer (IL-17A/A) consists of IL-17RA and IL-17RC (IL-17RA/RC) [4]. Additionally, the IL-17F/F homodimer and IL-17A/F heterodimer bind to the IL-17RA/RC heterodimer. IL-17RA/RD is an alternative receptor for IL-17A/A, but not for IL-17F/F or IL-17A/F. Recently, L-17RC/RC has been identified as another receptor for IL-17A/A, IL-17F/F and IL-17A/F. IL-17A/A has a high affinity for IL-17RA, whereas IL-17-A/F and IL-17F/F have an intermediate and low affinity, respectively. The affinity for IL-17RA decreases even more for IL-17B, C, D, and E. However, the affinity of IL-17F/F for IL-17RC is at least 40 times higher than for IL-17R [4].

Generally, responses to IL-17A signalling are stronger than those to IL-17F, which may account for the predominant role of IL-17A in promoting autoimmunity. Moreover, the expression profiles of the two chains of the IL-17 receptors differ: IL-17RA is expressed more prominently in the immune compartment and IL17RC primarily in nonimmune cells. It is unclear whether the various biological activities of IL-17A and IL-17F are caused by different expression profiles along with the different affinities of each receptor chain for IL-17A or IL-17F [3].

Different members of the IL-17 family come from various cellular sources. IL-17A, IL-17F, IL-17C, and IL-17E perform a variety of yet not fully known roles mediating inflammation in autoimmune, allergy, and chronic inflammatory disorders in addition to functioning in host defence against infections at epithelial interfaces [3].

IL-17A and IL-17F produced by different cell types belonging to the innate and adaptive immune systems are summarized in Table 1.

Th17 cells, the best-known and main source of IL-17A and IL-17F, depend on IL-23 to sustain their differentiation status. The binding of IL-23 to its receptors on the cell surface triggers a signalling cascade leading to the expression of ROR-gamma t transcription factor, the hallmark of Th17 differentiation, and eventual synthesis of IL-17A and IL-17F. Th17 differentiation requires TGF-β, IL-6, IL-1, or IL-21, whereas IL-23 is crucial for lineage survival and maintenance. IL-1 and IL-23 expand the populations of both IL-17F+ and IL-17A+ human CD4+ T cells. Both IL-17A and IL-17F are able to increase inflammation when combined with other cytokines, such as TNF-α, and pro-inflammatory mediators such as IL-6, IL-8, and CXCL1 can be secreted by fibroblasts and epithelial cells when IL-17A and IL-17F are present [14,15].

Other than Th17 cells, some cell types belonging to the innate immune system, such as innate lymphoid cells, also secrete IL-17 isoforms. Innate cells such as gamma delta (γδ) T cells, alpha beta (αβ) T cells, type 3 innate lymphoid cells (ILC3), natural killer T (NKT) cells, and mucosal associated invariant T (MAIT) cells can release IL-17A and IL-17F regardless of IL-23 stimulation in certain circumstances and play crucial roles in inflammation and autoimmune disease. Thus, the relationship between IL-23 stimulation and the production/release of IL-17 isoforms is non-linear, and the roles of IL-17A and IL-17F may differ in different tissues, possibly explaining why IL-23 suppression appears to be of no therapeutic benefit on axSpA [6,11]. Furthermore, mast cells are able to capture and release IL-17A contributing to local tissue inflammation, and neutrophils might exert a similar role [16,17,18,19].

**Table 1:** Summary of immune cells that produce IL-17A and IL-17F.

	PsA	axSpA	Enthesitis	References
CD4+ helper T cells (Th17)	Increased number of Th17 in blood and synovial fluid compared to healthy controls.	Increased number compared to healthy controls.	Produce IL-17A when stimulated.	[20,21,22,23]
CD8+ cytotoxic T17	Increased frequency of CD8+ T cells in blood and synovial fluid.	Found in blood, digestive tract, and synovial fluid of patients.	Produce IL-17A when stimulated.	[21,22,23,24,25,26,27,28,29,30,31]
γδ T cells	Elevated frequency in synovial fluid of patients with active arthritis.	Increased number in blood and synovial fluid.	Physiologically present in entheseal tissue. Able to produce IL-17A without IL-23 stimulus.	[11,21,23,32,33,34,35,36,37,38]
MAIT cells	Proportionally, higher levels found in joints than in peripheral blood.	Elevated number compared to healthy controls in blood and synovial fluid.	Physiologically present in blood and healthy entheses.	[7,8,10,39,40,41]
IL-17+ Natural Killers T cells	Present in synovial fluid.	Increased levels in synovial fluid but might play an anti-inflammatory role in joints.	Unknown.	[21,37,38,39,42]
Group 3 innate lymphoid cells	Enriched in blood and synovial fluid, which might correlate with disease activity.	Elevated levels found in blood, digestive tract, bone marrow, and synovial fluid.	Physiologically present in entheses and adjacent bone.	[21,23,37,43,44,45,46]
Natural Killers	Present in synovial fluid, although in lower concentrations than in rheumatoid arthritis.	Increased number of IL-17 producing cells compared to healthy controls in peripheral blood.	Unknown.	[47,48]

## 4. Physiological Activity of IL-17

IL-17A and IL-17F evolved to protect against infection via the regulation of protective responses against infections at mucosal and epithelial surfaces, including the intestines, skin, lungs, and oral cavity. The stimulation of the synthesis and release of molecules that promote epithelial barrier function, such as beta-defensins, S100 proteins, and lipocalin-2 (Lcn2), is crucial to the major role of IL-17A and IL-17F in regulating protective immunity [2,3,37,40]. For instance, Lcn2 competes with bacterial siderophores for free iron, preventing bacterial growth. They also contribute to the inflammatory environment that produces more cytokines, chemokines, and matrix metalloproteinases (MMP) [3]. These elements facilitate the activation and recruitment of immune cells to the infection site and strengthen defence against the invasive pathogens, with neutrophil accumulation as a foremost example [12]. Thus, stromal, innate, and adaptive immune cells work together under the control of IL-17A and, to a lesser extent, IL-17F. In several studies and models, the impairment of IL-17A and IL-17F has been shown to facilitate infection by different pathogens such as *Klebsiella pneumoniae*, *Francisella tularensis*, *Pseudomonas aeruginosa*, *Citrobacter rodentium*, *Candida albicans*, or *Staphylococcus aureus* [3]. Interestingly, the protective role of IL-17 against *Candida* infections seems to be more important in the oral mucosa than in the vaginal epithelium [49].

## 5. IL-17: Pathogenic Role in PsA and axSpA

Excessive IL-17 activity drives an increase in inflammatory and tissue-remodelling molecules that can result in tissue damage. This is consistent with the elevations in the serum and tissue levels of IL-17A, IL-17F, and other isoforms of IL-17 in a variety of autoimmune and immune-mediated inflammatory diseases (IMIDs), such as lupus, PsO, PsA, multiple sclerosis, rheumatoid arthritis (RA), axSpA, scleroderma, and hidradenitis suppurativa (HS) [3].

Axial spondyloarthritis, PsA, and reactive arthritis belong to the diverse group of immunological conditions known as seronegative spondyloarthritides. The axSpA spectrum includes radiographic axSpA (r-axSpA), also known as ankylosing spondylitis, defined by structural damage to the sacroiliac joints seen on plain radiographs, and non-radiographic(nr)-axSpA, where patients have no radiographic evidence of sacroiliitis. Mechanical strain is probably a significant environmental risk factor that interacts with different genetic components to cause disease. Spondyloarthritis is defined by new bone formation at the entheseal organ, where ligaments link onto bone; this pathomechanism stands in stark contrast to RA, where inflammation is associated with bone erosion and destruction, but not bone formation [5,11,50].

Matrix metalloproteinases MMP1, MMP9, and MMP13 are produced by target cells when IL-17A is present in inflammatory arthritis, which promotes the breakdown of the extracellular matrix in the joint. Additionally, the production of the receptor activator of nuclear factor-B (NF-B) ligand (RANKL; also known as TNFSF11) by osteoblasts can be increased by IL-17A, which can then activate osteoclasts and cause bone loss. Moreover, IL-17A promotes angiogenesis, which boosts blood flow and makes it easier for inflammatory cells to enter an inflamed joint [15].

Recent research has shown that STAT3-dependent osteoblast-mediated bone remodelling is mostly caused by the presence of IL-23R+ entheseal resident cells and their production of IL-17 and IL-22 [5].

While most of the available evidence on the pathogenic role of IL-17 cytokines in PsA and axSpA is focused on IL-17A, recent studies have addressed the effect of IL-17F. Glatt et al. have detected an expression of IL-17A and IL-17F mRNA in the synovial tissue of PsA patients, supporting a potential contribution of IL-17F to the immunopathology of PsA [51]. Both IL-17A and IL-17F increase the production of IL-8 and IL-6 when synoviocytes are stimulated in the presence of TNF, IL-17F being less potent than IL-17A; furthermore, the dual neutralisation of IL-17A and IL-17F produces a greater suppression of synoviocyte and primary normal human dermal fibroblast activation than the blockade of IL-17A or IL-17F [51].

In their study, Hymowitz et al. treated porcine and human articular cartilage explants with a range of IL-17F concentrations, and proteoglycan release and synthesis were measured [13]. In both systems, IL-17F induced significant cartilage matrix release and inhibited new cartilage matrix synthesis in a dose-dependent manner, in the same order of magnitude as that of the catabolic cytokine IL-1α. However, the ability of IL-17F to directly control cartilage matrix turnover varies depending on the species examined and may be related to receptor affinity. IL-17F could, in a dose-dependent way, promote IL-6 production in porcine and human articular cartilage. Particularly, IL-17F and IL-17A both produced IL-6 at doses where no appreciable change in matrix synthesis or turnover was seen in human cartilage. In contrast to the comparable potency on human cartilage matrix turnover, IL-17F was less potent than IL-17A in both porcine and human cartilage in terms of IL-6 production [13].

The levels of IL-17 and/or IL-23 are noticeably elevated in serum from axSpA and PsA patients [15,42,47]. Synoviocytes from PsA patients had greater levels of IL-17 receptor A (IL-17RA) expression, according to flow cytometry and Western blot analyses [15]. The upregulation of the IL-23/IL-17 pathway seems to be a significant risk factor for the development of axSpA, but the exact mechanism by which IL-23 causes disease is yet unknown [15,42,47]. As in the case of PsO and IBD, predisposition to AS and PsA is linked to genetic variations in the IL12B region, which encodes the IL-12 p40 component shared by IL-12 and IL-23; PsA and PsO are also linked to a variation in the IL23A region that encodes the IL-23 p19 subunit. Additionally, susceptibility variations in the IL23R locus, which codes for the IL-23 receptor, are linked to IBD, PsA, AS, and PsA [15].

Observations in murine models—where either TNF-α or IL-23/17 pathway dysregulation results in inflammation that extends to nearby synovium and bone—support the idea that enthesitis plays a significant role in the pathophysiology of axSpA. This mouse entheseal disease was initially demonstrated to be caused by a poorly characterised entheseal resident population of γδT cells that were the main source of IL-17A as a result of IL-23 overexpression [52]. The production of IL-17A, IL-17F, and IL-22 may also be induced in the absence of IL-23 by this highly plastic subset of T cells [11]. Similarly, human MAIT cells can produce IL-17A and IL-17F in the absence of IL-23 stimuli [6,10]. Interestingly, IL-17F is the dominant isoform produced by all innate lymphocytes, including MAIT cells, γδT cells, and ILC3s. These findings suggest that IL-17F and IL-17A production from MAIT cells may cause tissue inflammation without the involvement of IL-23, partially explaining the therapeutic gap between IL-17 and IL-23 targeting in some inflammatory disorders [6].

The literature describes a hierarchy in inflammatory potential, with IL-17A homodimers eliciting the strongest inflammatory response, followed by IL-17A/F heterodimers, and then IL-17F homodimers; several reports have demonstrated that IL-17F functions similarly to IL-17A, though with less potency [15]. On the other hand, blocking both IL-17A and IL-17F simultaneously in human Th17 supernatants is more successful at lowering the release of pro-inflammatory mediators from target cells than blocking IL-17A alone [15]. The hitherto underappreciated proinflammatory role of IL-17F—in addition to IL-17A—in PsO and PsA has been confirmed by the fast response and high levels of clearance observed in clinical trials with bimekizumab, a dual IL-17A and IL-17F neutralising antibody [14]. Even though IL-17A has a stronger pro-inflammatory effect than IL-17F at equal concentrations, the lesional skin and serum of patients with PsO contain greater amounts of IL-17F (up to 30-fold) [14]; on the other hand, the relative concentrations are reversed in peripheral spondyloarthritis joints [53]. Elevated serum levels of IL-17F have also been found in several other IMIDs, including PsA, axSpA, and HS [14].

In a small number of investigations, immunohistochemical analysis has been used to demonstrate the expression of IL-17F in RA and PsA synovial tissue in comparison to osteoarthritis samples; moreover, a recent study found that six out of 14 PsA synovial tissue samples had detectable levels of IL17F mRNA [14].

Burns et al. demonstrated that IL-17A and IL-17F are differently regulated and that high-strength T cell stimulation induces CD4+ T cells that produce IL-17F. The pro-inflammatory properties of IL-17F are potentiated by TNF-α [14]; for instance, the IL-17-induced synthesis of IL-6 and IL-8 by synovial fibroblasts (cell lines derived from RA and PsA patients) is enhanced when TNF-α is present. Their blocking tests showed that the combined IL-17A and IL-17F blockade is more efficient at lowering IL-6, IL-8, CXCL1, CXCL5, and CCL20 production by fibroblasts compared to the blockade of IL-17A alone, but the IL-17F blockade alone had no effect on cytokine secretion [14].

Regarding the expression of IL-17 subtypes, activated Th17 cells can be classified into subpopulations that express IL-17A, IL-17A/F, or IL-17F. The cytokine environment, the intensity or concentration of antigenic signals transmitted by the T cell receptor (TCR), and the length of the stimulus have all been found to regulate this differentiation [54,55]. Whereas high-strength stimulation favours the IL-17F+ subset of Th17 cells, low-strength T cell activation preferentially promotes the induction of the IL-17A+ subpopulation. Additionally, the cytokine profiles of IL-17A+ and IL-17F+ Th17 cells are different from one another. IL-17F+ cells express less IL-10 and GM-CSF and more IFN-γ than IL-17A+ cells, so they have been linked to a more pathogenic phenotype in inflammatory disorders [54].

Remarkably, the regulation of IL-17A and IL-17F expression varies across time. IL-17A is quickly generated following the stimulation of Th17 cells, whereas the expression of IL-17F rises gradually, reaching greater levels at later stages of activation. On the other hand, unlike IL-17F, IL-17A expression is not maintained by ongoing T cell activation. This may imply that whereas IL-17A is significant at the beginning of inflammation, IL-17F would become more significant as the inflammation progresses. This would explain why, in some circumstances, inhibiting IL-17A only could not be sufficient for a long-term disease control strategy and why combined inhibition of IL-17A and IL-17F might be preferable instead [56,57,58,59,60,61,62].

The principles underpinning the pathophysiological role of IL-17F in PsA and axSpA and the basis for its therapeutic targeting are summarised in Table 2. 

## 6. IL-17F Blockade and Therapeutic Considerations

IL-17A has been identified as a driver of joint and skin inflammation, and a preclinical promise of anti-IL-17A inhibitors has ripened into therapeutic success for diseases such as PsO, PsA, and axSpA. Complete remission is still uncommon, especially in rheumatologic patients, and many of them benefit only partially or not at all from therapy that inhibits these cytokines. Hence, dual cytokine inhibition, as opposed to a pure IL-17A blockade, might have a more significant effect on chronic tissue inflammation. Complete clearance rates of PsO in clinical trials with dual IL-17A/F inhibitors are higher than those achieved with IL-17A antagonists [63]. In vitro, combined neutralisation of IL-17A and IL-17F determined a higher inhibition of the activation of synoviocytes and primary normal human dermal fibroblasts than an individual blockade of IL-17A or IL-17F [51]; clinical confirmation of the potential implications of these findings in rheumatological diseases would require head-to-head clinical trials.

### 6.1. Bimekizumab

Bimekizumab is a humanised monoclonal IgG1 antibody that selectively neutralises both IL-17A and IL-17F and has been approved or is being evaluated for a variety of immune-mediated conditions, including PsO, PsA, and axSpA. This mechanism of action enables a more thorough suppression of disease inflammation than IL-17A inhibition alone through a larger reduction in gene expression, inflammatory cell migration, and generation of proinflammatory cytokines [51,64]. Further, unlike the IL-17RA inhibitor brodalumab, bimekizumab has no effect on IL-17E, which is known to have anti-inflammatory properties [65]. Furthermore, brodalumab does not inhibit signalling through the IL-17RC/RC homodimeric receptor for IL-17A, IL-17A/F, and IL-17F [3,4].

Several phase III clinical trials have demonstrated the superiority of bimekizumab over placebo, ustekinumab, adalimumab, and secukinumab in PsO, as summarized in Table 3 [63,65].

#### 6.1.1. Psoriatic Arthritis

Concerning PsA, the primary endpoint for the phase IIb dose-ranging, placebo-controlled BE ACTIVE trial was the American College of Rheumatology’s ACR50 response criteria at 12 weeks, which is more stringent than the ACR20 endpoint commonly used for PsA trials. Bimekizumab had a quick onset of effect: the first indications of improvement were confirmed at week 8, and by week 12, they were firmly established. At 12 weeks, compared with the placebo group, significantly more patients in the 16 mg bimekizumab (odds ratio [OR] 4.2 [95% CI 1.1–15.2]; *p* = 0.032), 160 mg bimekizumab (8.1 [2.3–28.7]; *p* = 0.0012), and 160 mg (loading dose) bimekizumab (9.7 [2.7–34.3]; *p* = 0·0004) groups achieved ACR50 response [66]. These outcomes held true regardless of prior use of TNF inhibitors [65,67]. At a dose of 160 mg of bimekizumab every 4 weeks, a high percentage of patients maintained similar levels of response for at least 3 years: at week 152, according to non-responder imputation analysis, 52.9% of patients achieved ACR50, 57.7% achieved PASI100 response, and 51.5% reached minimal disease activity (MDA) [68].

In BE OPTIMAL, a 52-week, randomised, double-blind, placebo-controlled with an active reference (adalimumab) phase III trial, bimekizumab demonstrated greater efficacy to placebo across the spectrum of PsA in patients who had not previously received biologic therapy. At week 16, the primary endpoint of ACR50 had been achieved: patients receiving bimekizumab (189 [44%] of 431) reached ACR50 response vs. placebo (28 [10%] of 281; odds ratio 7.1 [95% CI 4.6–10.9], *p* < 0.0001); the ACR50 response rate for the reference adalimumab group was 46% (64 out of 140) [69]. Similarly, ACR20 and ACR70 responses at week 16 were higher than in the placebo group (ACR20: 268 [62%] of 431 vs. 67 [24%] of 281, adalimumab 96 [69%] of 140; ACR70: 105 [24%] of 431 vs. 12 [4%] of 281, adalimumab 39 [28%] of 140) [69].

The responses to bimekizumab persisted over a 24-week period: 282 (65%) of 431 patients receiving bimekizumab had ACR20, 196 (45%) of 431 had ACR50, and 126 (29%) of 431 had ACR70 response. Moreover, at week 24, 122 (56%) of 217 patients with concurrent PsO and PsA had achieved complete skin clearing (PASI100), and 73% (158/217) had attained PASI90 [69].

Clinical responses were quick: for ACR20, the separation between the bimekizumab and comparator groups after a single dose of bimekizumab was seen as early as week 2 for ACR20 (117 [27%] of 431 vs. 22 [8%] of 281), and at week 4 for all ACR response criteria (ACR20: 182 [42%] of 431 vs. 37 [13%] of 281; ACR50: 76 [18%] of 431 vs. nine [3%] of 281; ACR70: 27 [6%] of 431 vs. one [<1%] of 281) [69]. A quick response was also seen in patients who switched from placebo to bimekizumab at week 16; their clinical outcomes had improved by week 24—eight weeks after their initial dose of bimekizumab—with ACR20, ACR50, and ACR70 responses of 175 (62%) of 281, 101 (36%) of 281, and 53 (19%) of 281, respectively [69].

In BE COMPLETE, a multicentric, phase III, double-blind, placebo-controlled, randomized trial, bimekizumab was tested in patients with active PsA and previous inadequate response or intolerance to TNF inhibitors. The primary endpoint—ACR50 at week 16—was achieved: [67] 116 (43%) of 267 patients receiving bimekizumab vs. 9 (7%) of 133 patients receiving placebo. ARC20 and ACR70 response rates were 67% (179/267) vs. 16% (21/133) and 27% (71/267) vs. 1% (1/133), respectively. Furthermore, among patients with PsO affecting at least 3% body surface area (BSA) at baseline, 121 (69%) of 176 patients achieved PASI90 response in the bimekizumab group vs. six (7%) of 88 patients who received placebo [67].

There is an ongoing study, BE VITAL, which evaluates the long-term efficacy and safety of bimekizumab on PsA.

On the 7th of June 2023, the European Commission granted marketing authorisation for BIMZELX® (bimekizumab) for the treatment of adults with active PsA. 

#### 6.1.2. Axial Spondyloarthritis

Regarding active AS, bimekizumab 160 mg every 4 weeks resulted in quick disease control compared to placebo in the phase IIb BE AGILE study. The 303 selected patients were randomised to bimekizumab 16 mg (*n* = 61), 64 mg (*n* = 61), 160 mg (*n* = 60), 320 mg (*n* = 61), or placebo (*n* = 60). At week 12, significantly more bimekizumab-treated patients achieved at least 40% improvement from baseline Assessment of SpondyloArthritis International Society (ASAS) scores (ASAS40) vs. placebo: 29.5%–46.7% vs. 13.3%; *p* < 0.05 all comparisons; OR vs. placebo 2.6–5.5 (95% CI 1.0 to 12.9). At week 48, 58.6% and 62.3% of patients receiving bimekizumab 160 and 320 mg throughout the study achieved ASAS40. At week 156, non-responder imputation analysis showed that 53.7% of patients (72.6% of observed cases) maintained ASAS40 responses [50].

The dual inhibition of IL-17A and IL-17F with subcutaneous bimekizumab 160 mg every 4 weeks resulted in significant improvements in ASAS responses, disease activity, physical function, pain, quality of life, and spinal mobility in patients with nr-axSpA and r-axSpA compared with placebo in BE MOBILE 1 (nr-axSpA) and BE MOBILE 2 (r-axSpA): two phase III, placebo-controlled, parallel, 52-week, clinical trials. The primary endpoint of ASAS40 responses at week 16 was met: nr-axSpA: 47.7% bimekizumab vs. 21.4% placebo; r-axSpA: 44.8% vs. 22.5%; *p* < 0.001. Moreover, ASAS40 response continued to increase to week 24 (nr-axSpA: 52.3%; r-axSpA: 53.8%) [50].

Additionally, the BE MOBILE trials show the efficacy of bimekizumab in treating patients with axSpA regardless of prior TNFi exposure [50]. ASAS40 response rates in TNFi-naïve patients (secondary endpoint of BE MOBILE 2) were 45.7% (84/184) vs. 23.4% (22/94) in patients with r-axSpA [50]. As in patients with r-axSpA, ASAS40 response rates at week 16 in TNFi-naïve patients with nr-axSpA were greater with bimekizumab (46.6% (55/118)) than placebo (22.9% (25/109)) [50]. Similarly, more patients with prior TNFi exposure achieved an ASAS40 response with bimekizumab vs. placebo at week 16 in both the nr-axSpA (60.0% (6/10) vs. 11.8% (2/17)) and r-axSpA (40.5% (15/37) vs. 17.6% (3/17)) populations [50].

An ongoing study, BE MOVING, evaluates the long-term efficacy and safety of bimekizumab on radiological and nr-axSpA in patients who participated in the BE MOBILE 1 and BE MOBILE 2 trials.

On the 7th of June 2023, the European Commission granted marketing authorisation for BIMZELX® (bimekizumab) for the treatment of adults with active axSpA, including nr-axSpA and ankylosing spondylitis, also known as r-axSpA. 

#### 6.1.3. Safety

Overall, bimekizumab has not demonstrated any unexpected safety findings. In the majority of its clinical trials, safety data were superimposable to those of anti-IL-17A drug studies, apart from an apparent higher risk of oral candidiasis of 15% in BE VIVID and 10% in BE READY vs. a rate of 1–4% in the placebo arms [63,65,68]. Adverse effect rates were similar in active and placebo groups for most of the available clinical trials—except for the ones mentioned beforehand—without reports of major adverse effects [63,65,68,70]. Nasopharyngitis, upper respiratory tract infection, and oral candidiasis were the most common adverse effects overall reported, which were mild or moderate [63,65,68,70].

### 6.2. Sonelokimab

Nanobodies, which are smaller molecules than typical monoclonal antibodies, may be more effective at penetrating tissue. Sonelokimab (M1095/ALX-0761) is a trivalent tandem nanobody that selectively binds to human IL-17F, human IL-17A and IL-17A/F, and human serum albumin (to extend plasma half-life); thus, it is a bispecific IL-17A and IL-17F inhibitor. Like bimekizumab, sonelokimab may be useful in treating PsA and axSpA. The improvement of arthritis and X-ray score in a preclinical model of induced rheumatoid-like arthritis in *Cynomolgus* monkeys suggests that this potential therapeutic agent may provide an alternative to suppress inflammatory responses in conditions such as PsA and axSpA. Nonetheless, further clinical trials are required to prove these hypotheses [64,71].

## 7. Conclusions

- Interleukin (IL)-17A and IL-17F are important cytokines in the IL-17 family that control both innate and adaptive immunity and are pathogenically relevant in several IMIDs [1,2,3,4,11,14];
- Responses to IL-17A signalling are stronger than those to IL-17F, which may account for the predominant role of IL-17A in inflammation in SpA and axSpA [3,15];
- Th17 cells, the best-known and main source of IL-17A and IL-17F, depend on IL-23 to sustain their differentiation status [14];
- Gamma delta T cells, αβ T cells, ILC3, NKT cells, and MAIT cells can release IL-17A and IL-17F regardless of IL-23 stimulation [6,11,52];
- IL-17F induces cytokine production that increases inflammation, angiogenesis, and tissue-remodelling that result in tissue damage in the peripheral and axial enthesis, bone, and cartilage [3,4,5,15,42,50];
- Blocking both IL-17A and IL-17F simultaneously is more successful at lowering the release of pro-inflammatory mediators from target cells than blocking IL-17A alone. Nonetheless, IL-17F the blockade alone has no effect on cytokine secretion [14];
- Contrary to IL-17A, the blockade of IL-17F might be beneficial against colitis, as evidenced in murine models [12];
- Elevated serum levels of IL-17F have been found in several IMIDs, including PsA, axSpA, and HS [15];
- The lesional skin and serum of patients with PsO contain greater amounts of IL-17F than IL-17A; on the other hand, the relative concentrations are reversed in peripheral spondyloarthritis joints [53];
- IL-17A seems involved in the induction of initial inflammatory injury, while IL-17F is probably responsible for its chronification [56,57,58,59,60,62];
- Bimekizumab is a bivalent monoclonal antibody that inhibits both IL-17A and IL-17F; it has been approved for PsO in adult patients and has proven its efficacy and safety in clinical trials on PsA, r-axSpA, and nr-axSpA [50,51,63,63,65,66,67,68,69];
- Sonelokimab is a nanobody-blocking IL-17A and IL-17F that has demonstrated efficacy in PsO; clinical trials on PsA are ongoing [64,71].

## Figures and Tables

**Table 2:** Cornerstones of the pathogenetic role of IL-17F and rationale of its therapeutic targeting in PsA and axSpA.

Via receptor binding, IL-17F activates transcriptional activation resulting in the production of proinflammatory cytokines, chemokines, and AMPs [3,4]; Increased levels of TNFα, IL-1, IL-6, G-CSF, GM-CSF, CXCL1, CXCL5, CCL2, CCL7, AMPs, and MMP, due to IL-17F stimuli, drive an increase in inflammation, angiogenesis, and tissue-remodelling that result in tissue damage in the peripheral and axial enthesis, bone, and cartilage [3,4,5,15,42,50]; Entheseal tissue, of high importance in the pathogenesis of PsA and axSpA, seems capable of producing IL-17F without IL-23 stimuli [6,11,52]; IL-17F might play a crucial role in the chronification of inflammation, whereas IL-17A might be responsible of the initial tissular noxa [56,57,58,59,60,61,62]; At the same concentrations, IL-17F signalling responses seem weaker than those of IL-17A. Synergy between the two has been demonstrated [14,15,53]; Dual blockade of IL-17A/F seems more potent than IL-17A blockade alone. Nonetheless, IL-17F blockade alone has no effect on cytokine secretion [14,15]; Blockade of IL-17F –as opposed to that of IL-17A– might be beneficial against colitis, as evidenced in murine models [12].

**Table 3:** Summary of clinical trials of bimekizumab and PsO [63,65].

Study	Duration	Branches	PASI90 Week 16	PASI100 Week 16
BE-VIVID	52 weeks	Bimekizumab	85%	59%
		Ustekinumab	50%	21%
		Placebo	5%	0%
BE-READY	56 weeks	Bimekizumab	91%	68%
		Placebo	1%	1%
BE-SURE	56 weeks	Bimekizumab	86%	61%
		Adalimumab	47%	24%
BE-RADIANT	48 weeks	Bimekizumab	86%	62%
		Secukinumab	74%	49%

## Data Availability

Data sharing is not applicable to this article as no new data were created or analysed in this study.
